# Supplementary material for: The interactome of intact mitochondria by cross-linking mass spectrometry provides evidence for coexisting respiratory supercomplexes
Source: Mol Cell Proteomics. 2017 Dec 8;17(2):216–32. doi: 10.1074/mcp.RA117.000470 (PMC5795388; doi:10.1074/mcp.RA117.000470)
Supplement: Supplemental Data [file supp_RA117.000470_133922_0_supp_23978_fzffwf.zip › spectra_annotation/mito_spectra_annotation/307-1-4-1-7-1.pdf]

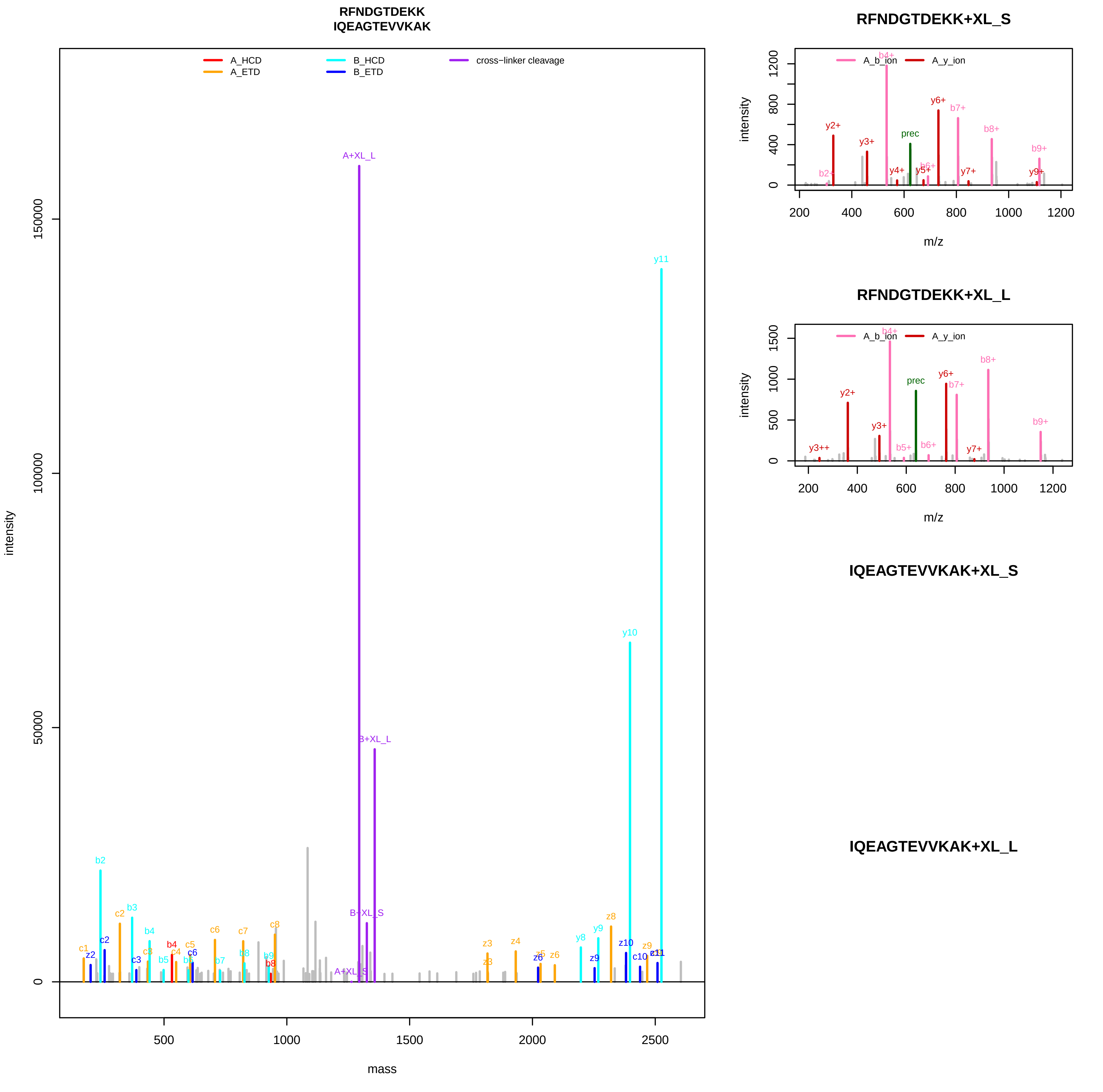

RFNDGTDEKK
IQEAGTEVVKAK
A_HCD
A_ETD
B_HCD
B_ETD
cross−linker cleavage
A+XL_L
B+XL_L
B+XL_S
A+XL_S
y11
y10
y9
y8
z8
z10
c10
z9
c11
z4
z3
z6
z5
z9
z6
b2
b3
b4
b5
b6
b7
b8
b9
c1
c2
c3
c4
c5
c6
c7
c8
z2
b4
b8
intensity
mass
0
50000
100000
150000
500
1000
1500
2000
2500
RFNDGTDEKK+XL_S
A_b_ion
A_y_ion
b2+
b4+
b6+
b7+
b8+
b9+
y2+
y3+
y4+
y5+
y6+
y7+
y9+
prec
intensity
m/z
0
400
800
1200
200
400
600
800
1000
1200
RFNDGTDEKK+XL_L
A_b_ion
A_y_ion
b4+
b5+
b6+
b7+
b8+
b9+
y3++
y2+
y3+
y6+
y7+
prec
intensity
m/z
0
500
1000
1500
200
400
600
800
1000
1200
IQEAGTEVVKAK+XL_S
IQEAGTEVVKAK+XL_L
